# The Institute of Medicine’s New Report on Living Well With Chronic Illness

**DOI:** 10.5888/pcd9.120126

**Published:** 2012-09-20

**Authors:** Jeffrey R. Harris, Robert B. Wallace

**Affiliations:** Author Affiliation: Robert B. Wallace, University of Iowa, Iowa City, Iowa.

In the United States, chronic illnesses such as heart disease, cancer, diabetes, stroke, and chronic lung disease account for 70% of deaths and 75% of health care costs (1,2) and have received attention in the professional and lay literature. Although efforts in managing chronic illness typically originate in the health care system, governmental and community-based public health organizations play an important role in helping people with chronic illness maintain optimal health. To help advance the chronic illness programs and policies of public health organizations, the Institute of Medicine (IOM), with the sponsorship of the Arthritis Foundation and the Centers for Disease Control and Prevention (CDC), has produced a new report, “Living Well With Chronic Illness: A Call for Public Health Action” (3). In this essay, we highlight findings from the report related to the consequences of chronic illness, the need for enhanced surveillance, the state of interventions and policies to decrease the effects of chronic illness, and the need for coordinated action in both health care and community-based settings. We close with a discussion of the report’s implications for public health organizations.

## The Consequences of Chronic Illness

In addition to highlighting the fatal consequences of chronic illness, the IOM report emphasizes their many nonfatal consequences. For example, 8.6 million Americans report living with disabilities related to arthritis (4). The consequences of chronic illness include myriad physical, mental, and social consequences that affect patients and their family members, friends, and caregivers. A person with arthritis may have all 3 consequences: physical, such as chronic pain; mental, such as depression; and social, such as an inability to work. Complicating our ability to track, treat, and manage chronic illness is the fact that commonly used outcome measures are not concordant for a given illness. Some illnesses, such as diabetes, have a great effect on care use, while others, such as arthritis, have their greatest effect on mobility, employment, and economic outcomes.

The IOM report highlights 9 chronic illnesses — arthritis, cancer survivorship, chronic pain, dementia, depression, diabetes, posttraumatic disabling conditions, schizophrenia, and vision and hearing loss. This list is not intended to indicate the most important chronic illnesses but is a means of illustrating their diverse sequelae, including emotional distress, sleep and pain symptoms, physical impairments, and age-related degenerative problems, all of which detract from living well. The report indicates that illnesses tend to cluster: among older adults, 43% have 3 or more illnesses (5) and 23% have more than 5 (6). The report also notes the disparities in chronic illness occurrence and care use according to race/ethnicity, income, and geography. For example, African Americans are twice as likely as whites to be diagnosed with diabetes (7).

## Surveillance, Interventions, and Policy 

Surveillance for chronic illnesses and their outcomes is critical to identifying needs and disparities, setting priorities for action, and assessing programmatic progress. The report recommends that surveillance be enhanced and that it be multilevel, multistage, and longitudinal. Levels are patient, health care system, population, and policy. Stages are the precursors of chronic illness, such as social determinants, biological risk factors, lifestyles, and receipt of evidence-based preventive interventions; illness occurrence and manifestations; and illness consequences, including physical, mental, and social. Longitudinal surveillance of chronically ill people will allow better assessment of both community-based and health care interventions, enabling more sophisticated analyses of what works. Supplementing current population surveys with information from electronic health records should produce more precise assessments of trends in improving quality of life for people living with chronic illness.

The intervention section of the report reinforces the preventive needs of people with chronic illnesses. In general, people who are chronically ill need all of the preventive services recommended for people who are not chronically ill, such as disease screening, immunizations, and lifestyle interventions to promote healthful eating, physical activity, smoking cessation, and weight maintenance. Preventive interventions for certain illnesses are paramount: for example, physical activity is important for people with arthritis to maximize their mobility and diminish disabling symptoms. Among lifestyle interventions, the benefits of physical activity for people with chronic illness are best documented. The IOM report cites physical activity trials that have shown decreased symptoms, improved functioning, or both in people with arthritis, cancer, depression, and diabetes (3), and more research is needed.

Public health programs and health systems need to promote community-based care, including chronic illness self-management and professionally driven disease management (eg, nurse help lines), cognitive training, and complementary and alternative medicine. There are promising reports for all of the community-based care methods, but more research is needed on how to adapt them to illnesses while meeting broad community goals cost-effectively.

Given the availability of both effective preventive interventions and effective community-based care, the next challenge is scaling up so that effective interventions reach all people in need, especially disadvantaged populations disproportionately affected by chronic illness. The IOM report calls for public health programs to be evaluated for their ability to reach people with chronic illness and deliver effective community-based interventions to them.

Public policies are critical to optimizing function and independence of the chronically ill, particularly those who are most disadvantaged in terms of income and disability. The report outlines decades of social policies and programs that lay a foundation on which to build, including support for income, medical care, and social services for the disabled, elderly, and vulnerable. One example is the Americans with Disabilities Act, which mandates accommodations for and nondiscrimination against people with disabilities. The Affordable Care Act (ACA), a more recent example, has broad implications for the chronically ill. For example, the ACA broadens health insurance coverage through Medicaid expansion, limits the impact of preexisting conditions on care costs, and promotes both coordination of care and preventive care. The report recommends a “health in all policies” approach that evaluates the effect on health and chronic illnesses of major policies in nonhealth sectors, such as agriculture, transportation, and housing. The report also calls for improved methods for economic evaluation of community interventions by both public health organizations and health care organizations.

## A Call to Action

Public health action to promote living well with chronic illness requires coordinated efforts in both health care and community-based settings. Most care for chronic illness occurs in health care settings. Because most of life happens outside of this realm, even for people living with chronic illness, there is great potential to leverage the infrastructure of community-based settings for both lifestyle interventions and community-based care. Many lifestyle programs have been developed and implemented in community-based settings such as worksites and by community-based service providers such as senior centers and YMCAs. These programs have often targeted the well rather than the chronically ill and have focused more on promoting healthy lifestyles than on community-based care. Policies and models that coordinate activities in both health care and community-based settings are largely untested (3). The IOM report suggests some advanced models that may support this coordination, but it also clarifies that none of the 3 major health care reimbursement systems — capitation, fee-for-service, or salary — provides adequate incentives for preventive and other care aimed at increasing quality of life for people with chronic illnesses. To realize the potential of these models, we need a realignment of incentives to reimburse service providers in community-based settings and an improved flow of communication and clinical information between health care and community-based settings.

## Implications

The IOM report reinforces the idea that the public health needs of the chronically ill are large, urgent, and growing and, therefore, have implications for governmental public health organizations, including CDC. In the 25 years since the creation of CDC’s National Center for Chronic Disease Prevention and Health Promotion, chronic illness prevention programs have become universal in state health departments (8) and have become more common, though not routine, in local health departments (9). These programs have focused most on cancer screening, although smaller disease-oriented programs for prevention and control of arthritis, diabetes, heart disease, and stroke have become common. Lifestyle programs for tobacco use control have also flourished but have been threatened by the recent economic downturn and the expiration and diversion of funds from the Master Settlement Agreement (10). Programs to promote healthful eating, healthy weight, and physical activity have been less well funded. ACA’s Prevention and Public Health Fund promises up to $2 billion per year for chronic illness prevention but is vulnerable to attempts to balance the federal budget. The underfunded state of chronic illness prevention programs begs the question of where governmental public health organizations and community-based health service providers will find the funds to add living well with chronic illness to their agendas. One answer is for them to collaborate more closely with health care organizations and share resources much more than in the past. The IOM report argues that they must.
